# ACD856, a novel positive allosteric modulator of Trk receptors, single ascending doses in healthy subjects: Safety and pharmacokinetics

**DOI:** 10.1007/s00228-024-03645-1

**Published:** 2024-02-14

**Authors:** Boel Nilsson, Johan Bylund, Magnus M. Halldin, Matthias Rother, Erik Rein-Hedin, Kristin Önnestam, Märta Segerdahl

**Affiliations:** 1 AlzeCure Pharma AB, Hälsovägen 7, SE-141 57 Huddinge, Sweden; 2 CTC Clinical Trial Consultants AB, Dag Hammarskjölds väg 10B, SE-752 37 Uppsala, Sweden; 3 Department of Surgical Sciences, Plastic Surgery, Uppsala University, SE-751 85 Uppsala, Sweden; 4 Department of Neurobiology, Care Sciences and Society, Karolinska Institute, Alfred Nobels allé 23, SE-141 52 Huddinge, Sweden

**Keywords:** Alzheimer’s disease, ACD856, Neurotrophin, First in Human, Single ascending doses

## Abstract

**Abstract:**

**Purpose:**

AlzeCure Pharma AB is developing novel positive allosteric modulators of Trk-receptors for treatment of Alzheimer’s disease, depression, other psychiatric conditions and other disorders where cognition is impaired. The preceding candidate drug ACD855 was shown to have a too long half-life in humans to allow further development. To de-risk the development of the follow-up compound ACD856, the oral single ascending dose study of ACD856 in humans was preceded by an intravenous microdose study, assessing the elimination half-life in plasma.

**Methods:**

A phase 0 study with a microdose of ACD856 (0.100 mg), was conducted in six healthy male subjects all receiving ACD856. Sequentially, a randomized, placebo-controlled, double-blind Phase I single ascending oral dose study (1 – 150 mg) was conducted, including 56 healthy subjects. Both studies assessed the safety and tolerability, as well as the PK properties of ACD856 after single dose intravenous and oral administration.

**Results:**

ACD856 was well tolerated with no treatment emergent, or dose related adverse events or other safety assessments. In the microdose study, ACD856 exhibited a bi-exponential plasma decline, low distribution volume, low plasma clearance with a half-life of approximately 20 hours. Orally, ACD856 exhibited rapid absorption, an almost complete bioavailability and a dose proportional increase in exposure. While the C_max_ was lowered and delayed by food intake, the effect on plasma half-life and the overall bioavailability was low. No renal elimination of ACD856 was detected.

**Conclusion:**

The prediction proved accurate demonstrating the value of conducting a microdose study prior to ascending dose studies.

**Trial registration:**

NCT05783830 March 24, 2023 (microdose study, retrospectively registered) and NCT05077631 October 14, 2021 (single ascending dose study).

**Supplementary Information:**

The online version contains supplementary material available at 10.1007/s00228-024-03645-1.

## Introduction

Dementia is one of the largest public health and social care challenges facing today’s society. Alzheimer’s disease is the most common neurodegenerative disorder resulting in progressive cognitive decline, affecting more than 50 million individuals globally, and expected to triple in the coming thirty years [1]. The current symptomatic therapeutics for Alzheimer’s disease (acetylcholinesterase inhibitors and memantine) have been shown to have limited efficacy and sometimes challenging side effects [2]. A systematic review and meta-analysis on the efficacy and safety of the available symptomatic treatments for patients with mild cognitive impairment showed that the current marketed products did not substantially improve function and cognition in this patient group and are associated with risks [2].

Aiming for disease modification by targeting the amyloid cascade has been the selected strategy for many drug development programs throughout the last 25 years [3, 6]. Encouraging clinical study data on the efficacy of antibodies targeting the amyloid cascade as a treatment for Alzheimer’s disease is emerging [4–6]. Aducanumab and lecanemab have already been granted approvals from the US Food and Drug Administration, and donanemab is expected to be granted approval in 2024. Although promising, it remains to be seen how widely these antibodies will be used in clinical practice. Even considering the availability of effective antibodies, a need for additional treatment options to manage Alzheimer’s disease remains. A new effective and safe symptomatic treatment with cognitive enhancing effects to be used alone or in combination with treatments such as the antibodies would be of great benefit to patients.

The importance of neurotrophin signaling pathways in the human brain has been well established. Pathways involving nerve growth factor (NGF) and brain-derived neurotrophic factor (BDNF) are of paramount importance for neuronal cell function, communication, and cell survival in brain areas vital for cognitive function, such as the hippocampus and basal forebrain. BDNF and NGF exert their effects through binding to their cognate tropomyosin receptor kinase (Trk) receptors: TrkA or TrkB, respectively [7, 8]. Pathological and mechanistic evidence suggests that loss of NGF signaling contributes significantly to the dysfunction of basal forebrain cholinergic neurons in Alzheimer’s disease [9, 10]. Decreased levels of BDNF have been observed in the hippocampus and in cerebrospinal fluid in disease states with cognitive decline, including Alzheimer’s disease [11–13], indicating that decreased BDNF signaling may contribute to this cognitive decline. The transplantation of stem cells or lentiviral delivery of BDNF into the brain of amyloid-transgenic mice or primates resulted in reversal of synapse loss and in improved cognition [14]. Furthermore, accumulating evidence suggests that increased BDNF signaling could improve cognition in Alzheimer’s disease [12, 15], indicating that positive modulation of NGF and BDNF signaling may be a relevant strategy to develop new treatment options for Alzheimer’s disease and other conditions where cognition is impaired.

The NeuroRestore^®^ program, initiated by AlzeCure Pharma AB, Sweden, aims to identify small molecules with a stimulatory effect on BDNF and NGF signaling. As reported by Dahlström et al. in 2021 [16], two lead molecules were identified as effective positive allosteric modulators of Trk receptors: ACD855 and ACD856. ACD855 is identical to the veterinary product Ponazuril, a triazine-based drug that acts to inhibit enzyme systems in protozoa and is used for the treatment of equine protozoal myeloencephalitis [17]. Both compounds demonstrated pro-cognitive effects in various preclinical in vivo models in mice [16, 18]. Cognitive enhancement was also seen in aged animals with a natural decline in memory, where a single dose of ACD856 led to an improved memory [16]. Furthermore, both ACD855 and ACD856 have also demonstrated anti-depressant-like effects in the forced swim test model [19].

The clinical efficacy, safety, and pharmacokinetics (PK) of ACD855 have been evaluated in various domestic animals for the use as a veterinary drug [20]. ACD855 was further investigated in general toxicity studies in rats and dogs with treatment duration of up to 28 days and assessment of recovery for 2 weeks, and there was no indication of any mutagenic or genotoxic potential. The PK of ACD855 in various animals indicated that ACD855 was rapidly absorbed, followed by a prolonged elimination, with terminal half-life in plasma between 0.3 to 7.9 days in animals. The elimination half-life in man was predicted through allometric scaling to 4.6 days. Based on pharmacology studies in rodents and safety studies in rat and dog, the first oral dose in an initiated Phase I single ascending dose (SAD) study (EudraCT No. 2018-002320-16), in healthy volunteers was set to 1 mg. After completion of cohort 1, the elimination half-life in plasma in humans was found to be significantly longer than predicted, with an average of 68 days (Figure S1). The study was prematurely terminated after interim PK analysis of the first cohort, and long-term follow-up of the dosed subjects was performed. The plasma exposure of ACD855 declined slowly over time, and all subjects but one still exhibited levels above the detection limit of 1 ng/mL at 280 days when the safety review committee decided that, in the absence of any safety concerns and the fact that the predicted AUC_inf_ was lower than the NOAEL in the most sensitive species, follow-up could be concluded.

The follow-on compound ACD856 was selected based on in vitro drug metabolism and PK properties, in addition to its demonstrated improved potency compared to ACD855. This included an improved profile with regard to metabolic characterization in hepatocytes and liver microsomes and in vivo PK in animals, predicting a shorter elimination half-life in humans.

This paper describes the start of the ACD856 clinical development program, including first drug administration in humans in an i.v. microdose study (NCT05783830) and an oral SAD study (NCT05077631). The aim was to evaluate the safety, tolerability, and pharmacokinetics following single doses of ACD856 in healthy subjects. In addition, the impact of food intake on PK properties was evaluated after oral dosing. Prior to initiating the microdose study, a limited program of toxicology studies was performed, in accordance with current guidelines and regulations. This included an evaluation of the genotoxic potential in silico and an extended single i.v. bolus dose toxicity study in rats. Additionally, due to notable clinical signs consisting of gastrointestinal disturbance in dogs with ACD855, a 7-day oral dose range-finding study in minipigs was conducted to assess the tolerability of ACD856 within this species. Prior to initiating the SAD study, ACD856 was tested in accordance with regulatory standards, including studies such as genotoxicity, general and safety pharmacology, and 28-day toxicology studies in rats and minipigs. No safety findings were found at the highest dose levels tested up to 28 days in both species (assessments included body weight, food consumption, ophthalmology, coagulation parameters, electrocardiogram (ECG), hematology, clinical chemistry, and urinalysis). An investigation into potential target-related adverse effect was done by reviewing publications from clinical studies of treatment approaches with similar modes of action, i.e., stimulating NGF or BDNF signaling [21–27]. In these studies, changes of gut motility (loose stools and increased frequency of stools), reduced appetite and weight loss, diffuse myalgia, and eosinophilia were observed as adverse effects potentially related to the mechanism of action. None of these adverse effects had been described as severe or serious adverse events.

## Methods

### Human ethics and consent to participate

The Phase 0 i.v. microdosing study was approved by the Swedish Medical Products Agency (MPA) (EudraCT 2019-003504-13, NCT05783830) and by the Swedish Ethics Review Authority (Dnr 2019-05317). All subjects gave their written informed consent before any study-related procedures were initiated. The Phase I oral single ascending dose study was approved by the Swedish Medical Products Agency (MPA) (EudraCT 2020-003379-16, NCT05077631) and by the Swedish Ethics Review Authority (Dnr 2020-04884). All subjects gave their written informed consent before any study-related procedures were initiated.

### Study design—Microdose study

Considering the results of the Phase I study of ACD855, an alternative strategy was selected when moving ACD856 into clinic, starting with a Phase 0 microdose study to gain an understanding of the human PK properties of the compound. Phase 0 approaches or so called “exploratory investigational new drug trials” are supported by available regulatory guidance [28, 29] and have successfully been used to minimize early attrition caused by inaccurate human PK prediction from preclinical data [30].

**Fig. 1:**
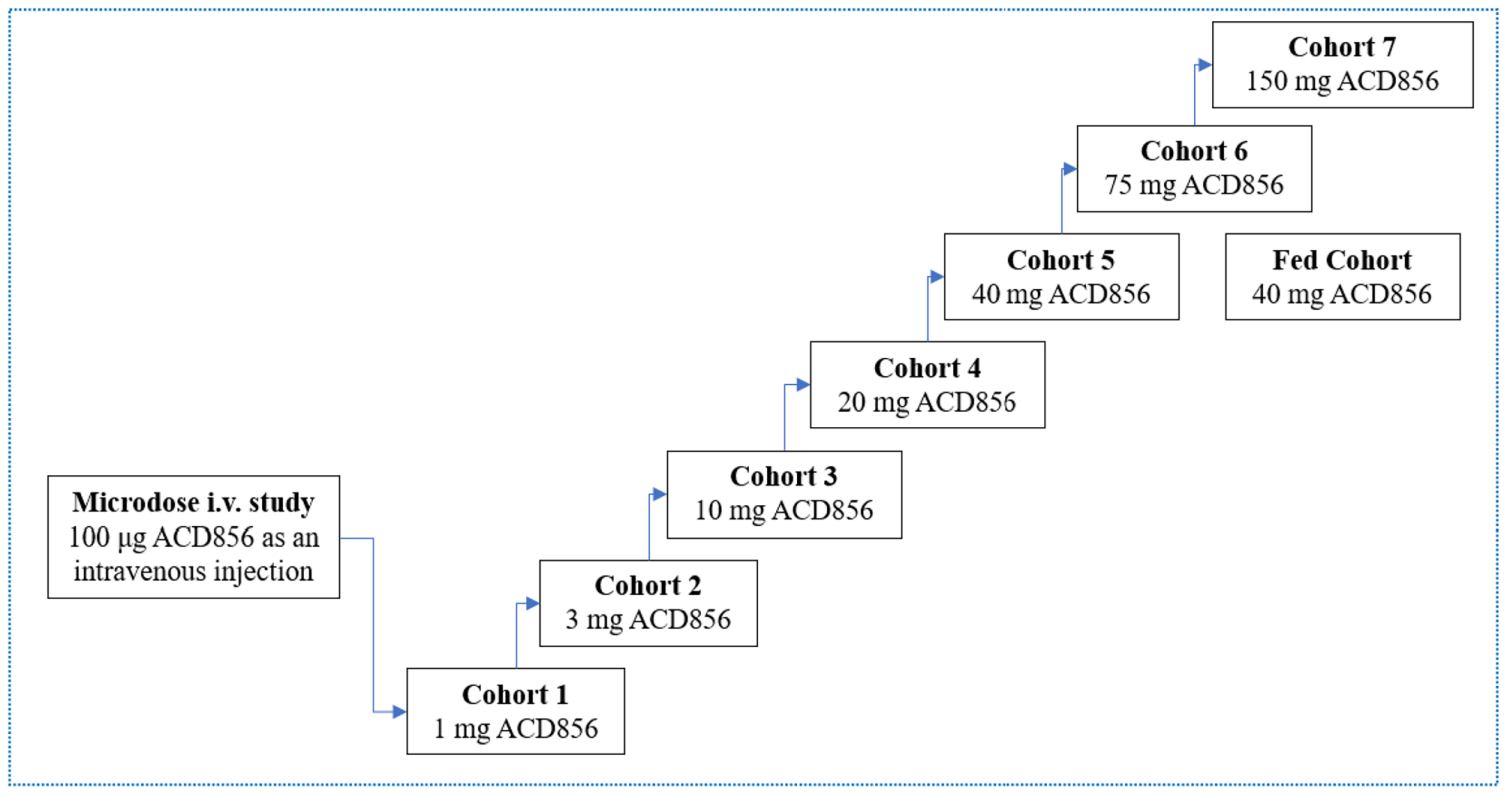
Illustrates the dose levels tested in each cohort of the two studies with ACD856 in humans; microdose i.v. and oral SAD study

**Fig. 2:**
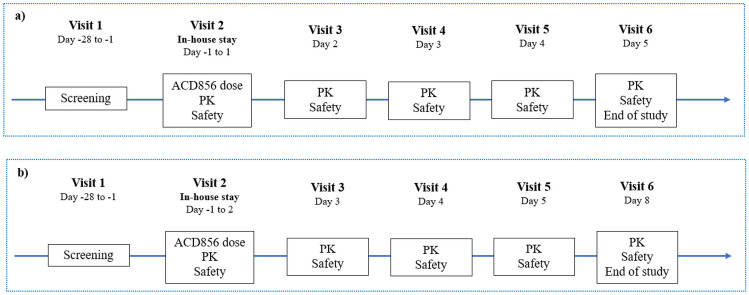
A schematic overview of the assessments performed at each visit in the two clinical trials of ACD856. **a** The microdose i.v. study. **b** The oral SAD study

The microdose i.v. study was an open-label, non-controlled study designed to evaluate the PK, safety, and tolerability of a single, bolus intravenous injection of a microdose of ACD856 in healthy male subjects (*n* = 6) (Figs. 1 and 2). Subjects in the study were administered 0.100 mg ACD856 as an i.v. injection following an overnight fast of at least 10 h. No food was allowed for at least 4 h post dose. Water, but no other drinks, was allowed as desired except for 1 h before and after ACD856 administration. Subjects in the i.v. study were dosed one by one in a sentinel fashion. The safety and PK properties observed from the first two subjects were evaluated prior to deciding on progressing to dose the remaining four subjects. There was sufficient time between dosing of each subject to allow for observation of any adverse reactions.

### Study design—Single ascending oral dose study

The SAD study was a double-blind, placebo-controlled randomized study evaluating the effects of single ascending oral doses of ACD856 on safety, tolerability, and PK in healthy subjects. ACD856 was administered in seven sequential cohorts, each consisting of 8 subjects, in which subjects were randomized to receive either ACD856 (*n* = 6) or placebo (*n* = 2) (Figs. 1 and 2). The observed PK properties of ACD856 in the microdose study were together with PK data generated in animal studies used for the predictions of human oral PK properties, and a suitable starting dose in relation to the estimated minimal anticipated biological effect level (MABEL) was calculated. The oral starting dose was 1 mg, and the additional evaluated doses were 3 mg, 10 mg, 20 mg, 40 mg, 75 mg, and 150 mg ACD856 or placebo (see Fig. 1). This cautious dose escalation strategy was selected as no target organ of toxicity had been identified in the preceding preclinical toxicology studies. Prior to dosing, the subjects fasted overnight for at least 10 h. No food was allowed for at least 4 h post dose. Water, but no other drinks, was allowed as desired except for 1 h before and after ACD856 administration. The food effect of a high-fat and high-calorie breakfast on the PK properties of ACD856 was investigated as part of the study. Subjects exposed to 40 mg ACD856 under fasting conditions returned to the clinic after a wash-out period to receive a second dose of ACD856 under fed conditions. In the fed cohort, subjects were served an FDA-standardized high-fat, high-calorie breakfast prior to dosing. The first two subjects in each SAD cohort were dosed in a sentinel fashion (one subject received ACD856 and one subject received placebo) in accordance with available guidance for First-in-Human clinical trials [31]. At least 48 h passed before dosing of the next subjects in the cohort to give sufficient time for observation of any reactions. The remaining subjects were dosed in groups of three and each group was dosed at least 24 h apart.

In both studies, the subjects were carefully monitored by clinical staff during and after dosing. Vital signs and ECG were checked at regular intervals. There was immediate access to equipment, qualified staff, and an intensive care unit in case of an emergency. After each dose level of the SAD study, the safety, tolerability, and PK of ACD856 were assessed by a Safety Review Committee which decided on escalation of the dose to the next cohort.

In the SAD study, the study medication was administered orally as a clear solution, with ACD856 (2 mg/mL) or the same contents without the active ingredient (placebo). The study medication was prepared by unblinded pharmacists at the site. All other site staff, study subjects, and monitor and sponsor personnel were blinded to study treatment allocation until database lock.

An overview of the study dose levels and cohorts is shown in Fig. 1.

### Study participants

For the microdose i.v. study, participation was restricted to male subjects, and for the SAD study, both male subjects and female subjects of non-childbearing potential were invited for enrollment. Study participants were eligible for inclusion if they were healthy, aged ≥ 18 and < 65 years, and with a body mass index between 18.0 and 30.0 kg/m^2^ at screening.

### Study assessments

Safety and tolerability assessments included adverse events (AEs), laboratory (clinical chemistry, hematology, coagulation, urinalysis), vital signs (systolic and diastolic blood pressure, pulse rate, and body temperature), 12-lead ECG, physical examination, and assessment of stool frequency. AEs were collected from the start of the study treatment until the end-of-study visit. Severity grading of AEs was done according to common terminology criteria for adverse events (CTCAE) v5.0 [32]. Causality of the AEs was assessed as whether there was a reasonable possibility that the event may have been caused by the study drug or not.

Blood samples and urine were collected at pre-defined timepoints in relation to study treatment throughout the studies for the determination of ACD856 concentrations and for the calculation of PK parameters. During the microdose i.v. study, blood samples were collected prior to dosing (0 h) and at 0.08, 0.17, 0.25, 0.5, 1, 1.5, 2, 3, 4, 6, 8, 10, 12, 24, 36, 48, 72, 96 and 120 h after dosing. In the SAD study, blood samples were taken prior to dosing (0 h) and at 0.17, 0.33, 0.5, 0.75, 1, 2, 4, 8, 16, 24, 32, 48, 72 and 96 h after dosing. The blood samples were centrifuged at 1500 G for 10 min to separate the plasma. The plasma samples were frozen within 1 h after collection and maintained frozen (at ≤  − 70 °C) until bioanalysis. Urine samples were collected on day 1 in the SAD study during the following intervals: pre-dose, 0–6, 6–12, and 12–24 h, and stored frozen (at ≤  − 70 °C) until analysis.

Plasma and urine samples were analyzed for exposure of ACD856 by Lablytica Life Science AB, Uppsala, Sweden. The validated bioanalytical method utilized extraction of ACD856 followed by ultra-performance liquid chromatography–mass spectrometry/mass spectrometry (UPLC‑MS/MS) analysis. The lower limit of quantification (LLOQ) of ACD856 in plasma was set to 0.1 ng/mL (i.v.) and 10 ng/mL (oral) and in urine to 100 ng/mL.

Figure 2a and b shows an overview of the study design and assessments.

### Pharmacokinetic and statistical analysis

No formal sample size calculations were performed for the microdose i.v. and oral SAD studies. The sample size was considered sufficient to provide adequate information for the study objectives as per current guidance. Safety and PK data were summarized by descriptive statistics. Dose proportionality was analyzed using linear regression modelling. All descriptive summaries and statistical analyses were performed using SAS Version 9.4.

The PK parameters were calculated by non-compartmental analysis using the software Phoenix WinNonlin^®^ version 8.1 or 8.3 (Certara, USA). Plasma concentrations below LLOQ were considered missing values for the microdose i.v. study. For the SAD study, LLOQ concentrations were set to 0 before t_max_ and to missing thereafter. The area under the curve (AUC) was calculated based on actual timepoints and according to the linear up-log down method. Measured PK parameters of ACD856 in plasma in the microdose study following i.v. bolus injection included initial plasma concentration (C_0_), elimination half-life (t_1/2(z)_), area under the concentration–time curve from zero to the time of the last concentration above the limit of quantification (AUC_0–last_), area under the concentration curve to infinity (AUC_0–inf_), plasma clearance (CL), and volume of distribution (V_z_ and V_ss_). Following oral administration in the SAD study, the following PK parameters were calculated: Maximum plasma concentration (C_max_), time to peak concentration (t_max_), elimination half-life (t_1/2(z)_), AUC_0–last_, AUC_0–inf_, apparent plasma clearance (CL/F), and the apparent volume of distribution (V_z_/F). Dose proportionality based on AUC_0–inf_ and C_max_ was assessed, the amount of renally excreted ACD856, as well as the relative bioavailability for fasted versus fed condition in the four subjects which were dosed under both conditions. The absolute bioavailability of oral doses in comparison to i.v. data was calculated.

## Results

### Study population

In the microdose i.v. study, the study population consisted of six white males with a mean age (standard deviation (SD)) of 33.7 (13.0) years and a mean BMI (SD) of 27.1 (1.6) kg/m^2^.

Fifty-six healthy subjects were enrolled and received study treatment in the SAD study. All enrolled subjects completed all study visits for the evaluation of single-dose administration of ACD856 in a fasted state. Three subjects from the 40 mg dose group chose to abstain from participating in the food interaction part, leaving five subjects (four on active ACD856 treatment and one on placebo) completing the fed cohort. The SAD study population consisted of 48 males and eight females with a mean age (SD) of 37.9 (12.7) years and a mean body mass index (BMI) (SD) of 24.2 (2.9) kg/m^2^. Forty-seven of the 56 subjects were white, and nine subjects were Asian. The masking of study treatment was considered successful, and no differences in taste or appearance were reported by study participants.

A summary of study participant characteristics for each treatment regimen in the microdose i.v. and the SAD studies are available in Table 1.

**Table 1:** Summary of study participants’ characteristics

		Phase 0 0.1 mg (i.v.) (*n* = 6)	Phase I 1 mg (*n* = 6)	3 mg (*n* = 6)	10 mg (*n* = 6)	20 mg (*n* = 6)	40 mg (*n* = 6)	40 mg food interaction (*n* = 4)	75 mg (*n* = 6)	150 mg (*n* = 6)	Placebo (*n* = 14)
Age (years)	Mean (SD)	33.7 (13.0)	38.5 (12.6)	39.2 (16.7)	35.3 (11.1)	33.0 (13.3)	44.0 (16.1)	49.0 (16.3)	31.8 (5.7)	29.8 (5.0)	43.9 (12.8)
Height (cm)	Mean (SD)	181.8 (2.2)	177.7 (11.9)	175.2 (9.0)	175.8 (12.0)	173.8 (5.3)	174.0 (6.5)	172.0 (7.4)	183.2 (9.2)	176.0 (4.9)	181.2 (11.5)
Weight (kg)	Mean (SD)	89.5 (6.6)	80.27 (11.83)	75.92 (6.31)	74.57 (7.93)	69.98 (8.23)	73.37 (11.87)	70.75 (8.83)	80.47 (10.99)	69.28 (7.12)	80.66 (14.51)
Sex	Female	0	1 (17%)	1 (17%)	1 (17%)	1 (17%)	1 (17%)	1 (25%)	0	0	3 (21%)
	Male	6 (100%)	5 (83%)	5 (83%)	5 (83%)	5 (83%)	5 (83%)	3 (75%)	6 (100%)	6 (100%)	11 (79%)

*n* total number of subjects, *SD* standard deviation

### Safety and tolerability of single doses of ACD856

Treatment with ACD856 was well tolerated in both the microdose and the SAD study, with no reports of serious AEs. No treatment-emergent or dose-related trends were observed for AEs, laboratory safety assessments, vital signs, 12-lead ECG, physical examination, or stool frequency. There was no indication of increasing AE frequency with increasing doses of ACD856 and no obvious difference in the AE reporting between subjects receiving active treatment and subjects receiving placebo.

In the microdose i.v. study, four subjects reported a total of eight AEs of which seven were judged as possibly related to treatment (see Supplementary Table S1). One subject reported four of the AEs of which two were of severe intensity according to CTCAE [32]. This subject, an active weightlifter, had transient elevated liver enzymes on one occasion 5 days after receiving the ACD856 microdose which were at the time judged by the Investigator to be possibly related to study treatment. The subject was asymptomatic and was not treated with any prior or concomitant medications.

In the SAD study, 31 out of 56 subjects reported a total of 62 AEs (see Supplementary Table S2). Twenty-four events (reported by 13 subjects) were assessed as possibly related to study treatment and were reported by subjects receiving ACD856. Out of these, two events were reported as being of moderate intensity, the others were of mild intensity: The most common AE was headache, which was reported by eight out of 56 subjects on 11 occasions. Headache was only reported by subjects receiving ACD856 in the dose range 1 to 40 mg ACD856, i.e., no episodes of headache were reported in the two highest dose groups (75 mg and 150 mg ACD856) or in the placebo group.

### Pharmacokinetics of single doses of ACD856

**Fig. 3:**
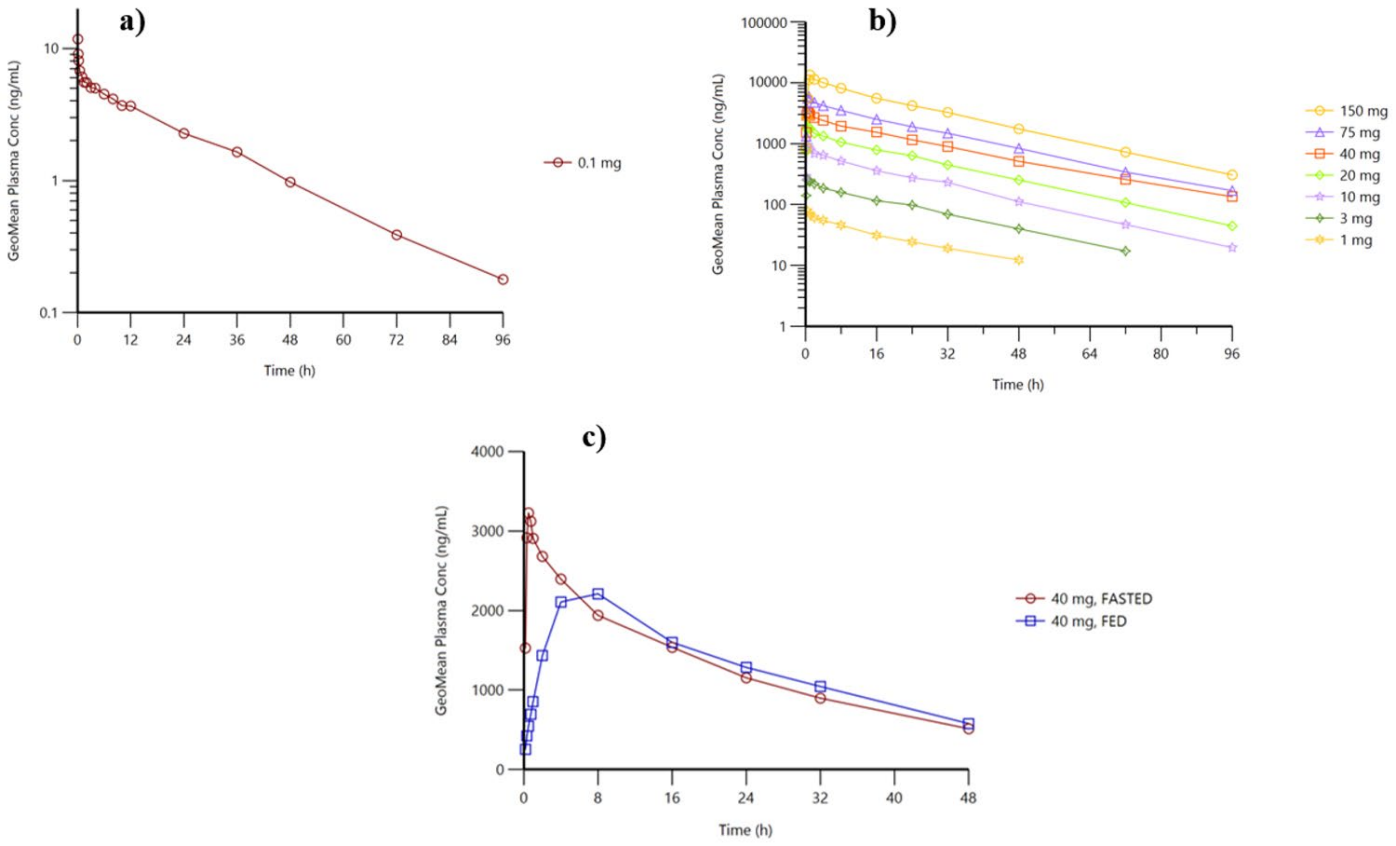
Mean plasma concentration curves for ACD856 following intravenous bolus (**a**), oral doses SAD study (**b**), and food interaction (**c**)

The mean initial plasma concentration (C_o_) following a single bolus i.v. injection of 0.1 mg ACD856 was estimated to be 15.4 ng/mL, and the subsequent decline in ACD856 plasma levels was bi-exponential (see Fig. 3). Both the volume of distribution (V_z_ of 17.6 L, and V_ss_ of 17.3 L (0.25 L/kg)) and the plasma clearance (CL of 0.64 L/h) were low (see Table 2). The mean terminal half-life of ACD856 was 19.4 h, with a mean AUC_0-inf_ of 160.9 h ng/mL.

**Table 2:** Summary of pharmacokinetic parameters of ACD856 following single doses of intravenous and oral administrations

**Parameter**	**Statistic**	**Phase 0 (i.v.) 0.1 mg**	**Cohort 1 (p.o.) 1 mg**	**Cohort 2 (p.o.) 3 mg**	**Cohort 3 (p.o.) 10 mg**	**Cohort 4 (p.o.) 20 mg**	**Cohort 5 (p.o.) 40 mg (fasted)**	**Cohort 6 (p.o.) 75 mg**	**Cohort 7 (p.o.) 150 mg**
**t**_**max**_	*n*	-	6	6	6	6	6	6	6
(h)	Median (Min, Max)	-	0.5000 (0.500, 1.00)	0.4167 (0.333, 2.00)	0.5000 (0.333, 1.00)	0.5000 (0.333, 0.767)	0.5083 (0.333, 0.750)	0.5000 (0.500, 0.750)	1.000 (0.750, 1.03)
**C**_**0**_	*n*	6	-	-	-	-	-	-	-
(ng/mL)	Mean (SD)	15.43 (2.633)	-	-	-	-	-	-	-
	Geometric mean (CV%)	15.25 (17.2)	-	-	-	-	-	-	-
**C**_**max**_	n	-	6	6	6	6	6	6	6
(ng/mL)	Mean (SD, CV%)	-	80.42 (13.26/16.5)	292.3 (24.35/8.33)	979.3 (81.34/8.31)	2047 (315.6/15.4)	3433 (708.4/20.6)	6327 (1035/16.4)	13430 (1264/9.41)
	Geometric mean (CV%)	-	79.58 (15.6)	291.5 (8.29)	976.4 (8.55)	2026 (15.6)	3366 (22.8)	6259 (16.0)	13380 (9.37)
**AUC**_**0-last**_	*n*	6	6	6	6	6	6	6	6
(h ng/mL)	Mean (SD^c^, CV%)	154.9 (30.00)	1295 (358.2/27.7)	5766 (681.7/11.8)	18460 (2147/11.6)	42150 (12820/30.4)	83160 (21360/25.7)	137500 (28800/21.0)	288600 (28500/9.87)
	Geometric mean (CV%)	152.5 (19.6)	1255 (27.8)	5732 (11.9)	18350 (12.2)	40640 (29.9)	80940 (25.7)	134900 (21.9)	287400 (10.2)
**AUC**_**0-inf**_	*N*	6	6	6	6	6	6	6	6
(h ng/mL)	Mean (SD^c^, CV%)	160.9 (30.18)	1711 (305.3/17.8)	6241 (755.2/12.1)	19030 (2286/12.0)	43130 (13050/30.3)	84560 (21150/25.0)	139200 (29170/21.0)	290300 (29020/10.0)
	Geometric mean (CV%)	158.6 (19.0)	1689 (17.6)	6202 (12.5)	18910 (12.6)	41590 (29.9)	82450 (24.8)	136600 (21.7)	289100 (10.3)
**t**_**1/2(z)**_	*N*	6	6	6	6	6	6	6	6
(h)	Mean (SD^c^, CV%)	19.37 (2.960)	19.93 (2.177/10.9)	20.36 (2.134/10.5)	19.07 (1.885/9.89)	19.67 (4.335/22.0)	23.48 (4.258/18.1)	21.37 (4.867/22.8)	19.62 (3.417/17.4)
	Geometric mean (CV%)	19.18 (16.0)	19.83 (11.0)	20.26 (10.8)	18.99 (9.75)	19.34 (19.5)	23.18 (17.1)	20.93 (22.8)	19.40 (16.2)
**CL/F**^a^	*N*	6	6	6	6	6	6	6	6
(L/h)	Mean (SD^c^, CV%)	0.6400 (0.1201)	0.5994 (0.1013/16.9)	0.4870 (0.06267/12.9)	0.5324 (0.06930/13.0)	0.4978 (0.1393/28.0)	0.4970 (0.1161/23.4)	0.5600 (0.1234/22.0)	0.5212 (0.05464/10.5)
	Geometric mean (CV%)	0.6306 (19.0)	0.5920 (17.6)	0.4837 (12.5)	0.5288 (12.6)	0.4809 (29.9)	0.4852 (24.8)	0.5492 (21.7%)	0.5189 (10.3%)
**Vz/F**^b^	*n*	6	6	6	6	6	6	6	6
(L)	Mean (SD^c^, CV%)	17.58 (2.312)	17.28 (3.665/21.2)	14.16 (0.8608/6.08)	14.57 (1.732/11.9)	13.60 (2.399/17.6)	16.44 (2.757/16.8)	16.73 (2.350/14.1)	14.61 (1.734/11.9)
	Geometric mean (CV%)	17.45 (13.4)	16.94 (22.6)	14.14 (6.09)	14.49 (11.5)	13.42 (18.1)	16.23 (18.2)	16.58 (14.8)	14.52 (11.7)
**V**_**ss**_	*N*	6	-	-	-	-	-	-	-
(L)	Mean (SD^c^, CV%)	**17.28** (2.403)	-	-	-	-	-	-	-
	Geometric mean (CV%)	**17.14** (14.2)	-	-	-	-	-	-	-

*AUC *area under the plasma concentration–time curve, *AUC*_*0-inf*_ AUC over the time infinity, *AUC*_*0-last*_ AUC over the time of the last measured concentration, *C*_*o*_ initial plasma concentration, *CL/F *apparent oral clearance, *C*_*max*_ maximum observed plasma concentration, *CV%* percent coefficient of variation, *n* total number of subjects, *SD* standard deviation, *t*_*1/2(z)*_ terminal elimination half-life, *t*_*max*_ time to C_max_, *V*_*z*_*/F *apparent volume of distribution associated with the terminal phase, *V*_*ss*_ apparent volume of distribution at steady state

^a^For the Phase 0 microdose i.v. study, the results refer to clearance (CL) and not oral clearance (CL/F)

^b^For the Phase 0 microdose i.v. study, the results refer to the volume of distribution associated with terminal slope (Vz), not the apparent volume of distribution (Vz/F)

^c^For the Phase 0 microdose i.v. study, the standard deviation (SD) is given

Following administration of an oral solution, the absorption of ACD856 was rapid in the dose range tested (1 to 150 mg) and reached maximum plasma concentration (C_max_) between 30 min and 1 h after administration under fasting condition (Table 2). Like the i.v. microdose cohort, both the volume of distribution (mean V_z_/F ranging between 13.6 and 17.3 L) and the plasma clearance (CL/F of 0.49–0.60 L/h) were low. The calculated terminal plasma half-life was approximately 20 h.

The plasma concentration profiles for the SAD study dose levels exhibited a bi-exponential decline, and the shape of the curves was similar for all dose groups where ACD856 was administered as single doses in the fasted state (Fig. 3). ACD856 exhibited a dose-proportional increase in plasma C_max_ and AUC_0-inf_ in the 1 to 150 mg dose range. The inter-individual variation (CV%) for all PK parameters within the same dose group was low, and the plasma exposure over the first 24 h (AUC_0-24_) was approximately 55% of AUC_0-inf_ (Table 2).

The time to reach maximum plasma concentration was considerably delayed following fed conditions consisting of a high-fat and high-calorie breakfast. The observed mean C_max_ under fed conditions was approximately 40% lower than the C_max_ observed under fasting conditions (see Supplementary Table S3). The calculated terminal plasma half-life was approximately 22 h under fed conditions, and the relative bioavailability for fed versus fasted was 93% (see Supplementary Table S3). Thus, the impact of food on the terminal plasma half-life and the overall bioavailability was low.

The oral bioavailability of ACD856 was estimated by comparing data from the microdose i.v. study and the oral cohorts of the SAD study. The observed geometric mean plasma clearance (CL) after intravenous administration of 0.1 mg ACD856 was 0.63 L/h. In the oral cohorts, the observed geometric mean apparent plasma clearance (CL/F) ranged between 0.48 and 0.59 L/h. These observations suggest that the oral bioavailability of ACD856 is almost complete.

All collected urine samples showed ACD856 concentrations below the detection limit (LLOQ) of the method, and hence, the amount of ACD856 eliminated renally could not be calculated. However, an estimation based on the collected urine volumes from subjects in the highest dose group (150 mg) and the LLOQ value (100 ng/mL) suggests that the eliminated amount of ACD856 during the first 24 h after dosing was less than 246 µg, which corresponds to less than 0.2% of the administered 150 mg dose. Thus, renal elimination of ACD856 appears to be non-existing or negligible.

## Discussion

ACD855 and ACD856 are both positive allosteric modulator compounds of Trk receptors, with ACD856 currently in clinical development for the treatment of Alzheimer’s disease, depression, other psychiatric conditions, and other disorders where cognition is impaired. Therapeutic development which is targeting the neurotrophin signaling pathways has been challenging due to their complex biology with an intricate pattern of multiple downstream signaling pathways [18]. ACD856 is being developed as a first-in-class drug candidate with a new mechanism of action to modulate the activity of the Trk receptors which may succeed in overcoming these challenges. It is hypothesized that ACD856’s positive modulating activity has advantages compared to an agonistic approach, leading to a more refined signaling pattern. Preclinical data gathered to date have been promising and have laid the foundation for the first clinical studies in humans described in this paper.

The development of the front-runner compound ACD855 was prematurely terminated after the completion of the first dose cohort (1 mg) in a SAD study conducted in healthy volunteers. The reason for the termination was that the observed plasma elimination half-life (68 days) was much longer than considered suitable for further clinical development. In order to de-risk the development of the follow-on compound ACD856, an alternate strategy was selected within the project. This entailed conducting an i.v. microdose study to evaluate the elimination half-life in plasma before initiating a comprehensive oral preclinical toxicity program and subsequently an oral clinical SAD study.

PK analysis of the i.v. microdose study clearly suggested that ACD856 had a much more suitable PK profile for clinical development than ACD855, and consequently, ACD856 was further evaluated in a SAD study in healthy volunteers following oral administration. The generated PK data from the i.v. microdose study was also used for predictions of ACD856 plasma concentrations following oral dosing as well as predictions of the starting dose of the oral SAD study.

No safety concerns were observed following ACD856 treatment in healthy subjects neither after the i.v. microdose nor after the oral single doses. No treatment-emergent or dose-related trends were observed for any of the safety parameters. This is in line with the lack of toxicology observations in preclinical studies in rats and minipigs. In the microdose i.v. study, one subject had a transient elevation of liver enzymes on one occasion 5 days after the ACD856 dose. Serology testing was done to rule out infection. Since then, substantially higher single and multiple doses of ACD856 have been administered to many healthy subjects in the SAD- and the subsequent multiple ascending dose (MAD) study (NCT05077501) [33] without any observations of treatment-emergent liver enzyme levels outside reference ranges. The subject in question was an active weightlifter who had been engaged in training just prior to lab assessments. Weightlifting has been shown to be associated with increased levels of liver enzymes [34]. Although the relationship to study treatment could not be ruled out at the time of study, upon retrospective review, the authors conclude that this finding most likely represents a sporadic event caused by intense physical activity. The study protocol and subject information sheet included restrictions to refrain subjects from strenuous activity during the duration of the study, which the subject did not adhere to.

Single oral doses of ACD856 showed a promising PK profile for further development. ACD856 was rapidly absorbed, demonstrated linear dose-dependent exposure, and exhibited a close to complete oral bioavailability. In addition, the observed terminal plasma half-life of approximately 20 h suggests once-daily dosing of ACD856 to be a suitable regimen in subsequent clinical development studies.

No renal elimination for ACD856 was detected. Thus, the metabolic excretion of ACD856 remains to be further explored.

The human oral clearance of ACD856 was approximately 70 times higher compared to the human oral clearance of ACD855, and is, as such, a great example of a successful optimization of a follow-up compound with regard to PK properties. The clinical investigations of ACD855 and ACD856 demonstrate the value of conducting a small microdose study in humans to receive information about a compound’s PK properties before making commitments to larger and more costly SAD/MAD studies.

It may be concluded from this case story that including microdose studies in a clinical development plan reduces the risk exposing trial subjects to a drug candidate with an unfavorable PK profile in a larger SAD study.

Administration of a single dose of ACD856 under fed conditions resulted in a considerably slower absorption rate and a lower C_max_ (40%) compared to fasted conditions, but the impact of food on the terminal plasma half-life and the overall bioavailability was low (see Table S3).

The main limitation of the current First-in-Human trials is their small study population, which is an inherent feature of such studies. The number of subjects per dose cohort is in agreement with available guidance for First-in-Human ascending dose studies [31] and is deemed sufficient to evaluate safety and pharmacokinetics with the purpose of proceeding to higher dose levels. Considering the limited sample size and that the studies had a single dose regimen, the results do not allow for general conclusions regarding the safety of ACD856; however, the results of these studies strongly support further clinical development with this drug candidate. The large number of single-dose cohorts included in the studies, ranging from 0.1 to 150 mg, adds to the strength and reliability of the study results. The number of subjects dosed with ACD856 in both fasted and fed conditions was small (*n* = 4), and while the results are promising as it indicates dosing flexibility in relation to food intake may be allowed, further studies of food interaction will need to be performed when the final formulation of the compound is available.

Data from current studies is a first small step in the clinical development of a new, effective, and safe treatment for patients with Alzheimer’s disease and other disorders where cognition is impaired, such as Parkinson’s disease and traumatic brain injury. Recent data have also indicated a potential for ACD856 for the treatment of depression and other psychiatric disorders [19]. ACD856 has been shown to be safe and well tolerated at the tested single-dose levels in healthy subjects. In addition, it has a suitable pharmacokinetic profile with a rapid absorption, linear dose-dependent exposure, and an almost complete bioavailability. In the next step, ACD856 has been evaluated in a multiple ascending dose study to evaluate the safety, PK, and pharmacodynamics of repeated doses in healthy subjects [33].

### Supplementary Information

Below is the link to the electronic supplementary material.

Supplementary file1 (DOCX 108 KB)

## Data Availability

The data that supports the findings of this study are available in the article and the supplementary material of this article.
