# Supplementary material for: ACD856, a novel positive allosteric modulator of Trk receptors, single ascending doses in healthy subjects: Safety and pharmacokinetics
Source: Eur J Clin Pharmacol. 2024 Feb 14;80(5):717–27. doi: 10.1007/s00228-024-03645-1 (PMC11001683; doi:10.1007/s00228-024-03645-1)
Supplement: Supplementary file 1 — Supplementary file1 (DOCX 108 KB) [file 228_2024_3645_MOESM1_ESM.docx]

**Supplementary Materials**

**Table S1: Reported adverse events by system organ class and preferred term for the microdose study**

|  | Total N=6 | |
| --- | --- | --- |
| System organ class  Preferred term | n(%) | m |
| **Respiratory, thoracic and mediastinal disorders** | **2(33%)** | **2** |
| Epistaxis | 1(17%) | 1 |
| Nasal congestion | 1(17%) | 1 |
| **Investigations** | **1(17%)** | **4** |
| Alanine aminotransferase increased | 1(17%) | 1 |
| Aspartate aminotransferase increased | 1(17%) | 1 |
| Blood creatine phosphokinase increased | 1(17%) | 1 |
| Blood lactate dehydrogenase increased | 1(17%) | 1 |
| **Musculoskeletal and connective tissue disorders** | **1(17%)** | **1** |
| Flank pain | 1(17%) | 1 |
| **Nervous system disorders** | **1(17%)** | **1** |
| Tremor | 1(17%) | 1 |
|  | | |

n, number of subjects; m, number of events

Percentages are based on the number of subjects in the treatment period included in the Full analysis set.

**Table S2: Reported adverse events by system organ class and preferred term for the single ascending dose study**

|  | 1 mg (N=6) | | 3 mg (N=6) | | 10 mg (N=6) | | 20 mg (N=6) | | 40 mg Fasted (N=6) | | 40 mg Fed (N=4) | | 75 mg (N=6) | | 150 mg (N=6) | | PLACEBO (N=14) | | Total (N=56) | |
| --- | --- | --- | --- | --- | --- | --- | --- | --- | --- | --- | --- | --- | --- | --- | --- | --- | --- | --- | --- | --- |
| System organ class  Preferred term | n (%) | m | n (%) | m | n (%) | m | n (%) | m | n (%) | m | n (%) | m | n (%) | m | n (%) | m | n (%) | m | n (%) | m |
| **Total** | **5 (83%)** | **14** | **4 (67%)** | **5** | **4 (67%)** | **9** | **2 (33%)** | **5** | **4 (67%)** | **6** | **3 (75%)** | **4** | **2 (33%)** | **6** | **4 (67%)** | **7** | **5 (36%)** | **6** | **31 (55%)** | **62** |
| **Gastrointestinal disorders** | **2 (33%)** | **2** | **1 (17%)** | **1** | **2 (33%)** | **2** | **0** | **0** | **2 (33%)** | **2** | **0** | **0** | **0** | **0** | **1 (17%)** | **1** | **2 (14%)** | **2** | **10 (18%)** | **10** |
| Abdominal discomfort | 0 | 0 | 0 | 0 | 0 | 0 | 0 | 0 | 0 | 0 | 0 | 0 | 0 | 0 | 0 | 0 | 1 (7.1%) | 1 | 1 (1.8%) | 1 |
| Abdominal pain | 0 | 0 | 0 | 0 | 0 | 0 | 0 | 0 | 1 (17%) | 1 | 0 | 0 | 0 | 0 | 0 | 0 | 0 | 0 | 1 (1.8%) | 1 |
| Abdominal pain upper | 0 | 0 | 1 (17%) | 1 | 0 | 0 | 0 | 0 | 0 | 0 | 0 | 0 | 0 | 0 | 0 | 0 | 0 | 0 | 1 (1.8%) | 1 |
| Diarrhoea | 0 | 0 | 0 | 0 | 0 | 0 | 0 | 0 | 1 (17%) | 1 | 0 | 0 | 0 | 0 | 1 (17%) | 1 | 0 | 0 | 2 (3.6%) | 2 |
| Nausea | 2 (33%) | 2 | 0 | 0 | 2 (33%) | 2 | 0 | 0 | 0 | 0 | 0 | 0 | 0 | 0 | 0 | 0 | 0 | 0 | 4 (7.1%) | 4 |
| Oral mucosal blistering | 0 | 0 | 0 | 0 | 0 | 0 | 0 | 0 | 0 | 0 | 0 | 0 | 0 | 0 | 0 | 0 | 1 (7.1%) | 1 | 1 (1.8%) | 1 |
| **Nervous system disorders** | **2 (33%)** | **4** | **1 (17%)** | **1** | **2 (33%)** | **3** | **1 (17%)** | **1** | **1 (17%)** | **1** | **3 (75%)** | **3** | **0** | **0** | **0** | **0** | **0** | **0** | **10 (18%)** | **13** |
| Headache | 2 (33%) | 4 | 1 (17%) | 1 | 1 (17%) | 2 | 1 (17%) | 1 | 1 (17%) | 1 | 2 (50%) | 2 | 0 | 0 | 0 | 0 | 0 | 0 | 8 (14%) | 11 |
| Somnolence | 0 | 0 | 0 | 0 | 0 | 0 | 0 | 0 | 0 | 0 | 1 (25%) | 1 | 0 | 0 | 0 | 0 | 0 | 0 | 1 (1.8%) | 1 |
| Tremor | 0 | 0 | 0 | 0 | 1 (17%) | 1 | 0 | 0 | 0 | 0 | 0 | 0 | 0 | 0 | 0 | 0 | 0 | 0 | 1 (1.8%) | 1 |
| **Psychiatric disorders** | **1 (17%)** | **2** | **1 (17%)** | **1** | **1 (17%)** | **1** | **0** | **0** | **0** | **0** | **0** | **0** | **1 (17%)** | **2** | **1 (17%)** | **2** | **2 (14%)** | **2** | **7 (13%)** | **10** |
| Abnormal dreams | 0 | 0 | 0 | 0 | 0 | 0 | 0 | 0 | 0 | 0 | 0 | 0 | 1 (17%) | 2 | 0 | 0 | 0 | 0 | 1 (1.8%) | 2 |
| Anxiety | 0 | 0 | 1 (17%) | 1 | 1 (17%) | 1 | 0 | 0 | 0 | 0 | 0 | 0 | 0 | 0 | 1 (17%) | 1 | 0 | 0 | 3 (5.4%) | 3 |
| Depressed mood | 0 | 0 | 0 | 0 | 0 | 0 | 0 | 0 | 0 | 0 | 0 | 0 | 0 | 0 | 1 (17%) | 1 | 0 | 0 | 1 (1.8%) | 1 |
| Middle insomnia | 1 (17%) | 1 | 0 | 0 | 0 | 0 | 0 | 0 | 0 | 0 | 0 | 0 | 0 | 0 | 0 | 0 | 1 (7.1%) | 1 | 2 (3.6%) | 2 |
| Nightmare | 1 (17%) | 1 | 0 | 0 | 0 | 0 | 0 | 0 | 0 | 0 | 0 | 0 | 0 | 0 | 0 | 0 | 1 (7.1%) | 1 | 2 (3.6%) | 2 |
| **Skin and subcutaneous tissue disorders** | **0** | **0** | **1 (17%)** | **1** | **2 (33%)** | **2** | **1 (17%)** | **2** | **0** | **0** | **0** | **0** | **1 (17%)** | **1** | **1 (17%)** | **1** | **0** | **0** | **6 (11%)** | **7** |
| Alopecia | 0 | 0 | 0 | 0 | 1 (17%) | 1 | 0 | 0 | 0 | 0 | 0 | 0 | 0 | 0 | 0 | 0 | 0 | 0 | 1 (1.8%) | 1 |
| Cold sweat | 0 | 0 | 0 | 0 | 1 (17%) | 1 | 0 | 0 | 0 | 0 | 0 | 0 | 0 | 0 | 0 | 0 | 0 | 0 | 1 (1.8%) | 1 |
| Pruritus | 0 | 0 | 1 (17%) | 1 | 0 | 0 | 1 (17%) | 2 | 0 | 0 | 0 | 0 | 0 | 0 | 0 | 0 | 0 | 0 | 2 (3.6%) | 3 |
| Rash | 0 | 0 | 0 | 0 | 0 | 0 | 0 | 0 | 0 | 0 | 0 | 0 | 0 | 0 | 1 (17%) | 1 | 0 | 0 | 1 (1.8%) | 1 |
| Rash papular | 0 | 0 | 0 | 0 | 0 | 0 | 0 | 0 | 0 | 0 | 0 | 0 | 1 (17%) | 1 | 0 | 0 | 0 | 0 | 1 (1.8%) | 1 |
| **General disorders and administration site conditions** | **2 (33%)** | **4** | **0** | **0** | **1 (17%)** | **1** | **1 (17%)** | **1** | **0** | **0** | **0** | **0** | **0** | **0** | **1 (17%)** | **1** | **0** | **0** | **5 (8.9%)** | **7** |
| Fatigue | 1 (17%) | 3 | 0 | 0 | 1 (17%) | 1 | 0 | 0 | 0 | 0 | 0 | 0 | 0 | 0 | 0 | 0 | 0 | 0 | 2 (3.6%) | 4 |
| Feeling hot | 1 (17%) | 1 | 0 | 0 | 0 | 0 | 0 | 0 | 0 | 0 | 0 | 0 | 0 | 0 | 0 | 0 | 0 | 0 | 1 (1.8%) | 1 |
| Medical device site reaction | 0 | 0 | 0 | 0 | 0 | 0 | 1 (17%) | 1 | 0 | 0 | 0 | 0 | 0 | 0 | 1 (17%) | 1 | 0 | 0 | 2 (3.6%) | 2 |
| **Musculoskeletal and connective tissue disorders** | **1 (17%)** | **1** | **0** | **0** | **0** | **0** | **0** | **0** | **1 (17%)** | **2** | **1 (25%)** | **1** | **1 (17%)** | **1** | **0** | **0** | **0** | **0** | **3 (5.4%)** | **5** |
| Arthralgia | 0 | 0 | 0 | 0 | 0 | 0 | 0 | 0 | 0 | 0 | 1 (25%) | 1 | 1 (17%) | 1 | 0 | 0 | 0 | 0 | 2 (3.6%) | 2 |
| Back pain | 1 (17%) | 1 | 0 | 0 | 0 | 0 | 0 | 0 | 0 | 0 | 0 | 0 | 0 | 0 | 0 | 0 | 0 | 0 | 1 (1.8%) | 1 |
| Pain in extremity | 0 | 0 | 0 | 0 | 0 | 0 | 0 | 0 | 1 (17%) | 2 | 0 | 0 | 0 | 0 | 0 | 0 | 0 | 0 | 1 (1.8%) | 2 |
| **Cardiac disorders** | **0** | **0** | **0** | **0** | **0** | **0** | **0** | **0** | **1 (17%)** | **1** | **0** | **0** | **0** | **0** | **1 (17%)** | **2** | **0** | **0** | **2 (3.6%)** | **3** |
| Atrioventricular block second degree | 0 | 0 | 0 | 0 | 0 | 0 | 0 | 0 | 1 (17%) | 1 | 0 | 0 | 0 | 0 | 0 | 0 | 0 | 0 | 1 (1.8%) | 1 |
| Ventricular tachycardia | 0 | 0 | 0 | 0 | 0 | 0 | 0 | 0 | 0 | 0 | 0 | 0 | 0 | 0 | 1 (17%) | 2 | 0 | 0 | 1 (1.8%) | 2 |
| **Infections and infestations** | **0** | **0** | **0** | **0** | **0** | **0** | **1 (17%)** | **1** | **0** | **0** | **0** | **0** | **0** | **0** | **0** | **0** | **1 (7.1%)** | **1** | **2 (3.6%)** | **2** |
| Nasopharyngitis | 0 | 0 | 0 | 0 | 0 | 0 | 1 (17%) | 1 | 0 | 0 | 0 | 0 | 0 | 0 | 0 | 0 | 0 | 0 | 1 (1.8%) | 1 |
| Oral herpes | 0 | 0 | 0 | 0 | 0 | 0 | 0 | 0 | 0 | 0 | 0 | 0 | 0 | 0 | 0 | 0 | 1 (7.1%) | 1 | 1 (1.8%) | 1 |
| **Respiratory, thoracic and mediastinal disorders** | **1 (17%)** | **1** | **0** | **0** | **0** | **0** | **0** | **0** | **0** | **0** | **0** | **0** | **1 (17%)** | **1** | **0** | **0** | **0** | **0** | **2 (3.6%)** | **2** |
| Oropharyngeal pain | 1 (17%) | 1 | 0 | 0 | 0 | 0 | 0 | 0 | 0 | 0 | 0 | 0 | 1 (17%) | 1 | 0 | 0 | 0 | 0 | 2 (3.6%) | 2 |
| **Ear and labyrinth disorders** | **0** | **0** | **1 (17%)** | **1** | **0** | **0** | **0** | **0** | **0** | **0** | **0** | **0** | **0** | **0** | **0** | **0** | **0** | **0** | **1 (1.8%)** | **1** |
| Vertigo | 0 | 0 | 1 (17%) | 1 | 0 | 0 | 0 | 0 | 0 | 0 | 0 | 0 | 0 | 0 | 0 | 0 | 0 | 0 | 1 (1.8%) | 1 |
| **Injury, poisoning and procedural complications** | **0** | **0** | **0** | **0** | **0** | **0** | **0** | **0** | **0** | **0** | **0** | **0** | **1 (17%)** | **1** | **0** | **0** | **0** | **0** | **1 (1.8%)** | **1** |
| Ear injury | 0 | 0 | 0 | 0 | 0 | 0 | 0 | 0 | 0 | 0 | 0 | 0 | 1 (17%) | 1 | 0 | 0 | 0 | 0 | 1 (1.8%) | 1 |
| **Investigations** | **0** | **0** | **0** | **0** | **0** | **0** | **0** | **0** | **0** | **0** | **0** | **0** | **0** | **0** | **0** | **0** | **1 (7.1%)** | **1** | **1 (1.8%)** | **1** |
| Blood creatine phosphokinase increased | 0 | 0 | 0 | 0 | 0 | 0 | 0 | 0 | 0 | 0 | 0 | 0 | 0 | 0 | 0 | 0 | 1 (7.1%) | 1 | 1 (1.8%) | 1 |
|  | | | | | | | | | | | | | | | | | | | | |

n=number of subjects; m= number of events

Individuals participating in the 40 mg food interaction group and one individual from the placebo group received treatment twice. These subjects are only counted once in the total. Percentages are based on the number of subjects in the treatment period included in the Full analysis set.

**Table S3: Summary of Observed Pharmacokinetic Parameters of ACD856 Following a Single Oral Administration of 40 mg in the fasted versus fed cohorts.**

| **Assessment (unit)** |  | **40 mg Fasted** | **40 mg Fed** |
| --- | --- | --- | --- |
| T_max_ (h) | n | 4 | 4 |
|  | Median (Min, Max) | 0.5000 (0.333, 0.517) | 6.000 (4.00, 8.02) |
| C_max_ (ng/mL) | n | 4 | 4 |
|  | Mean (SD/CV%) | 3758 (467.0/12.4%) | 2363 (247.2/10.5%) |
|  | Median (Min, Max) | 3660 (3300, 4410) | 2415 (2020, 2600) |
|  | Geometric Mean (geo CV%) | 3737 (12.1%) | 2352 (10.9%) |
| AUC_0-last_ (h*ng/mL) | n | 4 | 4 |
|  | Mean (SD/CV%) | 89940 (22760/25.3%) | 83360 (14550/17.5%) |
|  | Median (Min, Max) | 92290 (64700, 110000) | 88510 (62500, 93900) |
|  | Geometric Mean (geo CV%) | 87700 (26.7%) | 82310 (19.1%) |
| AUC_0-24_ (h*ng/mL) | n | 4 | 4 |
|  | Mean (SD/CV%) | 47450 (9553/20.1%) | 40860 (4552/11.1%) |
|  | Median (Min, Max) | 46490 (38500, 58300) | 41050 (35100, 46200) |
|  | Geometric Mean (geo CV%) | 46730 (20.3%) | 40660 (11.3%) |
| AUC_0-inf_ (h*ng/mL) | n | 4 | 4 |
|  | Mean (SD/CV%) | 91350 (22690/24.8%) | 84180 (14870/17.7%) |
|  | Median (Min, Max) | 92890 (66800, 113000) | 89610 (62800, 94700) |
|  | Geometric Mean (geo CV%) | 89170 (26.0%) | 83080 (19.4%) |
| CL/F (L/h) | n | 4 | 4 |
|  | Mean (SD/CV%) | 0.4598 (0.1180/25.7%) | 0.4885 (0.1011/20.7%) |
|  | Median (Min, Max) | 0.4429 (0.355, 0.599) | 0.4474 (0.423, 0.637) |
|  | Geometric Mean (geo CV%) | 0.4486 (26.0%) | 0.4815 (19.4%) |
| V_z_/F (L) | n | 4 | 4 |
|  | Mean (SD/CV%) | 15.89 (3.054/19.2%) | 15.69 (2.209/14.1%) |
|  | Median (Min, Max) | 16.35 (11.8, 19.1) | 16.50 (12.4, 17.3) |
|  | Geometric Mean (geo CV%) | 15.66 (20.7%) | 15.56 (15.2%) |
| T_½(z)_ (h) | n | 4 | 4 |
|  | Mean (SD/CV%) | 24.57 (5.037/20.5%) | 22.65 (3.900/17.2%) |
|  | Median (Min, Max) | 23.86 (19.4, 31.1) | 22.98 (18.3, 26.3) |
|  | Geometric Mean (geo CV%) | 24.19 (20.4%) | 22.40 (17.7%) |
| Relative Bioavailability | n | - | 4 |
|  | Mean (SD/CV%) | - | 0.9370 (0.1180/12.6%) |
|  | Median (Min, Max) | - | 0.9070 (0.833, 1.10) |
|  | Geometric Mean (geo CV%) | - | 0.9317 (12.2%) |
| Ratio C_max_ | n | - | 4 |
|  | Mean (SD/CV%) | - | 0.6362 (0.1061/16.7%) |
|  | Median (Min, Max) | - | 0.6309 (0.537, 0.745) |
|  | Geometric Mean (geo CV%) | - | 0.6295 (16.9%) |
|  | | | |

Data reported for subjects participating in both fasted and fed cohorts of 40 mg. n= number of subjects in treatment group. Mean and median values are based on n. n: Number of observations. SD: Standard deviation. CV%: Coefficient of variation. Geo CV%: Geometric coefficient of variation calculated using log-transformed standard deviation.

**Figure S1**


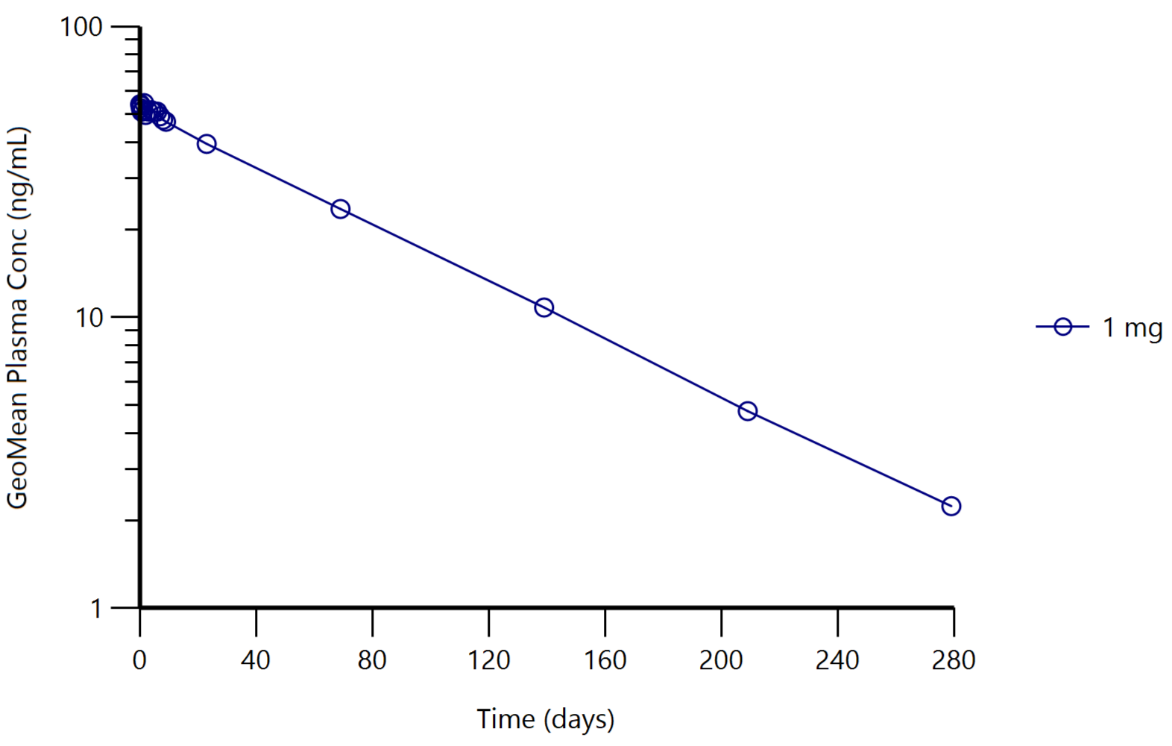


**Figure S1:** Mean plasma concentration curve for predecessor compound ACD855 following a single ascending oral dose of 1 mg.
